# Consultation etiquette in general practice: a qualitative study of what makes it different for lay cancer caregivers

**DOI:** 10.1186/1471-2296-12-110

**Published:** 2011-10-05

**Authors:** Letitia H Burridge, Geoffrey K Mitchell, Moyez Jiwa, Afaf Girgis

**Affiliations:** 1 Discipline of General Practice, School of Medicine, The University of Queensland, Level 8, Health Sciences Building, Royal Brisbane and Women's Hospitals, Herston, Queensland 4006, Australia; 2 School of Medicine (Ipswich), The University of Queensland, Level 4, Building 12, Salisbury Road, Ipswich, Queensland 4305, Australia; 3 Curtin University of Technology, Curtin Health Innovation Institute, Health Research Campus, Curtin University of Technology, GPO Box U1987, Perth WA 6845 Australia; 4 Translational Cancer Research Unit, Ingham Health Research Institute, Liverpool NSW 2170 Australia

## Abstract

**Background:**

It is commonplace for lay caregivers to overlook their own health concerns when supporting someone with advanced cancer. During this time, caregivers' needs as patients are often marginalised by health professionals, including General Practitioners (GPs), who may miss the breadth of caregivers' needs by focusing on the practicalities of caregiving. GPs traditionally rely on patients to raise their concerns, and then respond to these concerns, but caregivers as patients may be disinclined to cue their GP. The norms of engagement when caregivers consult their GP are less defined, and how they interact with their GP regarding their own health is under-explored. This sub-study investigates the norms, assumptions and subtleties which govern caregiver-GP consultations, and explores factors affecting their interaction regarding caregivers' own health concerns.

**Methods:**

We conducted semi-structured interviews with six lay caregivers and 19 health professionals in Brisbane, Australia, and analyzed the interview transcripts thematically.

**Results:**

Traditional norms of engagement are subjected to assumptions and expectations which caregivers and GPs bring to the consultation. Practice pressures also influence both parties' capacity and willingness to discuss caregivers' health. Nonetheless, some GPs monitor caregivers' health opportunistically. Their interaction is enhanced by the quality of the caregiver-GP relationship and by the GP's skills.

**Conclusions:**

Caregivers are caught in a paradox whereby their health needs may become subsumed by the care recipient's needs in a setting where patient needs are normally scrutinised and supported. Caregivers may not raise their health concerns with their GP, who instead may need to cue them that it is timely and safe to do so. The routine use of a prompt may help to address caregivers' needs systematically, but it needs to be complemented by GPs' desire and capacity to engage with patients in a caregiving role. The potential difference GPs can make to the health of these patients is substantial.

## Background

Cancer is a leading cause of morbidity and mortality [1,2]. The prevalence of cancer is rising with the ageing population [3], and increasing survival due to improvements in cancer detection and treatment [4]. At the same time, Australian families are growing smaller, which means a decrease in the traditional source of unpaid care, namely spouses and adult children in particular [3]. This scenario places greater pressure on family members to provide long-term support to cancer patients. Paradoxically, lay caregivers of advanced cancer patients function in a position of relative indifference toward their own health at a time when it is generally considered to be at increased risk.

Although many who fill the role are healthy, caregivers often have poorer than average health [5,6]. Several factors seem to contribute to this undesirable situation.

Firstly, caregivers tend to overlook their health. They perceive their own needs as insignificant compared to those of the person whose cancer is progressing [7], and they might not want to waste the doctor's time with their own "trivial" concerns [8]. Bruce *et al. *[9] explored what happened when caregivers did consult their General Practitioner (GP) and found that dementia caregivers tend to resist discussing their problems with the GP, making it difficult for the GP to gauge how well they are coping, and whether or when they should intervene. Even if they are aware of their health needs and willing to discuss them, caregiving responsibilities may make it difficult to attend their GP.

Secondly, GPs may overlook the unique needs of these patients. GPs tend to view caregiving as a practical role and risk missing its less tangible but important psychosocial and relational impacts [10]. Furthermore, GPs who care for both the patient and caregiver together may use a patient-centred approach with the cancer patient while unintentionally marginalising the needs of the caregiver as a patient, unless a separate appointment is made regarding their own specific health needs. In addition, some GPs may be unaware of the ambivalence which underlies the caregiving patient's reticence to raise such concerns, or their own potential role in validating the concerns of caregivers as patients. Fox *et al. *[11] found that GPs who had themselves experienced significant illness developed empathy for the vulnerability of being a patient, which in turn motivated them to put their own patients at ease by giving permission to raise any concerns.

Thirdly, the typically brief GP consultation makes it difficult for GPs to review the breadth of health concerns of caregivers as patients. This may be easier in an established caregiver-GP relationship, however, as the GP is often familiar with the patient and their life-context [12]. Insufficient GP training and remuneration is a significant barrier to preventive care that is oriented to physical diseases [13], and this may also be the case in the context of psychosocial preventive care. Whilst the ongoing care of cancer patients remains paramount, currently the implication is that the health of caregivers as patients is often a secondary concern. It is unclear whether GP-patient encounters routinely take these factors into account.

It is clear that being in a caregiving role complicates the relationship between caregivers as patients and their GP. Consultation styles are generally dominated by the biomedical model of illness, which assumes that illness is the consequence of disease [14]. In contrast, the patient-centred method enables non-biomedical factors that influence health to be considered in the consultation, which patients prefer [15]. A dual-agenda approach enables the patient to provide cues regarding their experience, while the GP's role is to encourage the patient to verbalise their concerns and then to *explain the illness *[16] (italics added), although it seems the agenda might still progress toward an illness-disease perspective. It may be difficult to adopt a holistic approach routinely in busy practices as it is potentially more time-consuming than the biomedical model [17]. Consultations are also problematic with patients in a caregiving role, as they often find themselves as the third person in a medical encounter focused, quite appropriately, on the cancer patient. Caregivers may also feel reluctant to disclose their own health concerns in front of the care recipient. When caregivers *are *able to attend their GP alone, it is assumed that they come with identified needs and a desire to resolve them, but caregivers may underestimate or even dismiss their needs at the very point where support is accessible. Like health professionals, lay caregivers can be drawn into the current of the "patient first" model of care which focuses on the cancer patient but simultaneously and inadvertently overlooks their own valid physical and non-physical health needs in challenging circumstances. Thus, GPs and their patients who are caregivers must negotiate around the limitations of the biomedical approach and the relative impracticalities of the patient-centred model to manage caregivers' health concerns. This creates additional communication challenges.

Underpinning this study are our beliefs that GP-based care of caregivers is achievable and desirable, and that two-way communication is fundamental to effective patient-doctor encounters. We assumed that the norms of patient engagement with GPs might be less certain for caregivers as patients because their caregiving role overshadows their own patient role.

### Aim and study question

In alignment with an ethnomethodological perspective [18], we examined the accounts of caregivers, GPs and other health professionals to understand how caregivers as patients make sense of their interactions with GPs regarding their own health, and do so in ways that seem socially acceptable [19].

Our research question was: "What do the views of lay caregivers and health professionals reveal about the way lay caregivers' health concerns are raised with their GP?"

## Methods

### Design

This qualitative study was conducted in May-October 2008 in Brisbane, Australia, as a sub-study of the GP Caregiver Toolkit project, a controlled trial whose aim is to develop and test an intervention to help lay cancer caregivers (hereafter "caregivers") proactively and systematically address their own health needs with their GP (ISRCTN43614355) [20]. This sub-study explored the views of current lay cancer caregivers, practising GPs and other health professionals regarding factors which influence how caregivers' health concerns are raised in the general practice setting. The project received ethical approvals from The University of Queensland and Princess Alexandra Hospital.

### Participants

A purposive sample of lay caregivers was recruited to gain useful insights into the research question from the often different experiences of men and women, older and younger caregivers, spousal and non-spousal caregivers, and those from urban and non-urban settings. We intended to cease participation when saturation of themes was reached if achievable within the 12-week recruitment period. Primary lay caregivers of patients with advanced cancer were recruited through medical personnel at the hospital oncology outpatient clinics. GPs and other key health professionals who care for cancer patients and their caregivers were recruited using a nationwide snowballing technique.

### Procedure

Potential participants were offered an information kit in person or by mail, and were followed up by phone after at least a week to answer any questions and ascertain their decision whether to participate. Participants were assured of anonymity and confidentiality of their data, and returned their voluntary consent by reply-paid mail or fax. In order to understand participants' perspectives, semi-structured in-depth interviews were conducted (by LB) with individual participants, using open-ended questions. These questions were developed with reference to the caregiving literature and published guidelines [19] to ensure clarity, consistency and quality, and focused on ability to manage the caregiving role and perceived health and well-being. Participants' views were sought regarding caregivers' key need and the potential benefit to caregivers of discussing their needs with their GP. All participants provided standard demographic information during their interview. The interviews lasted 45-60 minutes, were audio-recorded and transcribed verbatim by a professional stenographer.

### Analysis

The thematic analysis occurred in several steps [21]. First, two of the investigators (LB and GM) read each transcript line by line; then applied open coding to create a working list of descriptive categories which progressed toward saturation of themes; next we identified similarities and differences between and within the transcripts, noting patterns and exceptions, and recording our reflections on these. We noted interesting portions of text with reference to the research question, and explored ways in which these were related to the topic; then developed dimensions to group the descriptive concepts; and finally used axial coding to construct relationships between dimensions and concepts. We used quotations to illustrate the themes based on their capacity to provide clear descriptions for the interpretation, and to show overall patterns in the findings [19]. As knowledge is a social construct, our interpretation of the participants' accounts could be influenced by our own beliefs and assumptions. To counteract this, we looked for data to support alternative explanations while seeking a best-fit explanation of the data, and remained open to exceptions within the emerging patterns [19]. Two members of the team (LB and GM) conducted the analysis independently, and resolved any disparate views through discussion. By commencing the analysis during the data collection period, we were able to use the emerging themes to inform the direction of questions in subsequent interviews.

## Results

The demographic characteristics of the participants are presented in Table 1.

**Table 1:** Demographic characteristics of the participants

Characteristic	Caregivers (*n *= 6)	Health professionals (*n *= 19)
**Mean age, years (standard deviation)**	55.5 (17.1)	50.2 (5.6)
**Age groups**	**Category (n)**	**Category (n)**
< 50 years	2	8
50-64 years	2	11
≥65 years	2	0
**Gender**		
Women	5	7
Men	1	12
**Relationship to patient**		--
Spouse/partner	5	--
Non-spouse/partner	1	--
**Employment status**		--
Employed	3	--
**Profession**	--	
Practising GP	--	6
Palliative Specialist	--	5
Oncologist	--	2
Representative of caregiver/palliative care peak body	--	3
Policy personnel	--	2
Academic	--	1
**Practice location (GPs)**	--	
Metropolitan	--	3
Regional	--	1
Rural	--	2
**Years in position**	--	
< 10 years	--	8
≥10 years	--	11

Five of the caregivers were females. Four were solo caregivers, and two had dependent children; all had a regular GP, whom most visited at least four times per year (data not shown). The number of caregiver participants was smaller than expected. Site requirements meant we were dependent on busy medical specialists to confirm the eligibility of potential participants and refer them to project staff, who had no access to information about the number of caregivers approached, including all who were eligible, those who declined and their reason. Thirteen of the health professionals were medical practitioners, including six GPs and seven Palliative Care Medical Consultants. The remainder comprised two Registered Nurses (RNs), a Manager, an Educator, a Social Worker and a policy specialist.

Four themes around raising caregiver concerns were identified from the data: a) Inhibition resulting from traditional norms of engagement; b) Restraint arising from professional pressures; c) Communication enhanced by relational history; d) Constraint related to GPs' capacity and initiative.

Several abbreviations and notations are used in the quotations. The participant type and number are recorded in parentheses after quotations; for example, (HP1) signifies a comment made by the first health professional to be interviewed. The comments of GP participants are referenced using the symbol, "GP", to distinguish their views from those of other health professionals. For caregivers' comments, the symbol, "C" is used. Words omitted are indicated by "...", and editorial comments within quotations are enclosed in square brackets. Brief quotations are left within the text of the article and placed in quotation marks to distinguish them from the text.

### Theme 1: Inhibition resulting from traditional norms of engagement

There was little doubt among the participants that it would benefit caregivers to discuss their health concerns with their GP. However, there are barriers to this. Within an illness-disease framework, caregivers may see no genuine reason to engage with their GP. For example, it may simply not seem important enough, as "the majority...don't think to ask their GP" (HP11, Palliative Service Director). Preoccupation with the cancer patient alters caregivers' frame of reference, and they are likely to "forget, because there are that many other things going on in your mind" (C4). Alternatively, caregivers may consciously dismiss themselves as patients:

[Carers may feel] "we are not really legitimate players in this [but are] here to support our relative" (HP5, RN).

Some GPs may hold a similar view. One participant reasoned that his GP "is only interested in the [cancer] patient" (C5). This reveals a potential dilemma for caregivers, since

GPs put the patient first, which is appropriate to a point...[but they] have to clearly understand carers a lot more as patients in their own right (HP4, Educator).

Other GPs realise caregivers' propensity to trivialise their own health, noting that

They often feel so overwhelmed by what their [patient] is going through that anything they experience or feel they don't consider is important (GP3).

Unless caregivers reveal it, GPs may not recognise the protracted or demanding nature of caregiving or its potential toll on caregivers as patients. GPs respond differently to this situation. Some believe that the initiative remains the prerogative of the caregiving patient, and conclude that a caregiver who does not ask needs no help. One admitted his preference "to deal with [issues] as they come up" (GP18) while, in contrast, another GP endeavoured to discuss issues "at a fairly early stage and reinforce as need be...[because] if the GP doesn't facilitate it, people won't bring it up" (GP1). It may be problematic to do this, if caregivers have predetermined that their own health is not only irrelevant but also out-of-bounds. A GP's genuine inquiry may, paradoxically, produce an unappreciative response:

A lot of carers don't feel as though they are a priority or worthy of support. A lot of them think, 'It is my relative who is unwell. How dare [the GP] ask' (HP5, RN).

It seems that caregivers might permit the GP to inquire after the health of the cancer patient but not after the caregiver as a patient. Other HPs have encountered similar reactions [22]. This highlights the tightrope which the aware GP walks in order to convince caregivers that they are genuine patients with genuine health concerns. The participants' mixed views regarding who takes the initiative suggest that the norms which govern patient-doctor interaction [23] are themselves subjected to additional subtle conditions which the caregiving patient or GP bring to the encounter. Thus, caregiver health issues might remain hidden unless both GP and caregiver are able to negotiate beyond each other's norms of engagement and overcome the factors which may inhibit their interaction.

### Theme 2: Restraint arising from professional pressures

The participants identified several practice-oriented factors which influence the management of caregivers' health concerns. These include how GPs and caregivers perceive and address time constraints, and GPs' capacity to help caregivers.

Most caregivers felt sensitive to their GP's time constraints. Some were sympathetic and tried to avoid "wasting [the GP's] time" (C5). Besides subordinating their needs to the cancer patient, caregivers may fail to raise their health concerns for the GP's sake:

They think it is going to take too long. Patients are often very considerate of their doctors (HP13, Palliative Specialist).

When caregivers do wish to discuss their concerns with the GP, it is disappointing to have to wait weeks for an appointment, as by then, "the issue [on their mind] has probably gone" (C5). A younger caregiver admitted she was unlikely to discuss her own health with her GP as consultations seemed to be "rushed, or you are in and out" (C3). The GPs and other health professionals concurred with this perception and observed that "there are some GPs who, in this day and age, would decline to look after [caregivers]" (GP1). Even willing GPs are under pressure, and are often "...too rushed to be able to talk to people in detail about the fear or grief that they are feeling all through their caring journey" (HP4, Educator).

The problem is that this may reinforce caregivers' subordination of their own needs. It is also possible that caregivers' consideration for the GP's lack of time might conceal their deeper aversions, and they might stall on discussing difficult but valid issues. One GP remarked that caregivers

...don't like talking about [the caregiving situation]. It makes their grief surface. It makes them face up to the future. They don't want to "take up the GP's time" is usually the catch-cry and so you just have to sit back and say "I have got plenty of time. Hang what's happening out in the waiting room," or make another time. I find making another time is a bit fraught. A lot of them won't come back (GP1).

It seems that the alternative to prompt but brief access is to wait longer for an appointment. Both options may deter caregivers from raising their health concerns at all with the GP. To engage meaningfully with a caregiver, busy GPs must choose between limiting the length and scope of a consultation and opportunistically raising important issues which generate waiting room delays.

### Theme 3: Communication enhanced by relational history

The caregiver-GP relationship was an important factor which could enable or impede concerns being discussed. As a caregiver explained,

The GP would have to know the [caregiver]. If you dropped in...and saw someone [new] at a medical centre...you couldn't confide anything to them (C5).

An established relationship relaxes the norms of engagement, making it easier for caregivers to disclose their concerns, because the environment "is conducive to dealing with these things" (GP1). Ideally, this would give both GP and caregiver greater scope to raise issues when they seem important, but some caregivers reported that their regular GP was unreceptive. When asked if it would be beneficial for caregivers to discuss their needs with their GP, a 39 year old caregiver responded with an incisive counter-question: "What do you feel a GP would do for a carer?" (C3). It then became apparent that the caregiver did not feel this way about all GPs. Her long-standing previous GP "would do everything possible to try and help us out" (C3) but had moved too far away to visit, and she had been unable to find another who cared to the same extent. Rather than turn to her current GP regarding the impact of her husband's newly diagnosed aggressive cancer, the caregiver had phoned the former GP for guidance. This caregiver's re-initiation of contact with a GP by a long-distance phone call illustrates the extent to which the established, supportive relationship enabled her to deviate from consultation norms to deal with her concerns.

GPs who are aware of the caregiving context may defer to the caregiver to take the initiative, while making the effort to proactively cultivate a relationship which allows the opportunistic discussion of any issues that may need to be considered:

I have always been surprised at how well they cope and how little help they ask for....It is good if we don't need a lot of [other] services but it is important to establish a relationship. That allows a lot of these issues to be monitored and raised as they go along (GP3).

### Theme 4: Constraint related to GPs' capacity and initiative

GPs need well-developed communication skills to encourage caregivers to discuss their own health concerns. Nonverbal cues are important, from the caregiver point of view:

...body language would be very important....My GP...treats you like you are the most important person in the world when you are in the room with them (C4).

It was apparent to one GP that empathy and anticipation are acquired skills:

You do...better in your thirtieth year of practice perhaps than you did in your third. Unless you are dealing with a certain number of patients, then you won't have the competence or the confidence to do it and you certainly won't have the knowledge just for the way of getting the caregivers comfortable (GP1).

The participants identified several ways for GPs to indicate that this consultation is a safe opportunity for caregivers to discuss their concerns, and that it is useful to do so. For example, GPs could acknowledge their role in supporting the caregiver and make the environment comfortable for caregivers to disclose their concerns:

...acknowledging quite clearly that they have a role in supporting the family member as well, and outlining what that other support might entail (HP5, RN),

Making the environment comfortable and making sure they have some answers, if they can, or point in the right direction (HP14, Palliative Specialist),

...initiat[ing] the topic themselves by just asking how they are managing and how they are feeling and they could ask them like what things are difficult and raise it, give them some information about what resources are available (HP17, Palliative Specialist).

A GP can bring perspective to caregiver-relevant information because:

"Caregivers can be bombarded with support services. Knowing which one is the priority and how to outline them is the next step; I think that is important" (HP5, RN).

Such opportunities might be more easily found away from the demands of the practice environment, during house calls for example. This would require commitment however, as:

The one thing you can never fake in general practice consultation is the time you spend. It takes time to address these issues. It takes time to allow people the opportunity to think and bring areas up so I often find it happens much more readily in the home environment. I will go and see people at the end of the day in their own home, without time pressures of people waiting outside the door, hoping to get in. They are more likely to spend time to talk. Early-on discussion of issues, that process can take several visits and an hour or more each time (GP18).

GPs require a high level of communication skills and the commitment to find ways to engage with caregivers as patients proactively as well as reactively, in order to address their health concerns. Thus, the norms of engagement are also influenced, from GPs' perspective, by their capacity for the task.

## Discussion

We present new insights into the way caregivers as patients interact with their GP. Traditional norms of engagement and GPs' professional pressures restrain the discussion of caregiver health concerns, whereas interaction can be enhanced by a positive GP-patient relationship and by GPs' capacity to modify their consultation style with these patients. Our findings concur with others that patients might not cue their GP regarding their own health needs while caring for someone with cancer [8,24]. This study highlights the reality that GPs might not cue caregivers as patients either. That it is beneficial for caregivers to discuss their own health concerns with their GP did not seem to be in dispute for most participants in the present study. The primary issue is how to facilitate this where it would not otherwise happen. As in the context of dementia [9], cancer caregivers and their GPs may bring their own norms, assumptions and ambivalence to the consultation. This calls for mutual willingness to negotiate beyond the presenting problem, face each others' defences and conventions, and proactively attend to the health and well-being of caregivers as patients when it is important to take this initiative. Dealing with these things takes time. Caregivers as patients may lack the perspective to understand that proactively addressing their health is desirable, but even busy GPs are better placed than caregivers to rectify any misperception that proactive care for caregivers is a waste of GPs' time.

The literature suggests that tradition tends to guide consultations between hospital doctors and their patients [16,23]. The findings of this study suggest that the role of caregivers as patients during consultations with their GPs is less clearly scripted, both in content and frequency. When being proactive is more appropriate than the standard presenting-problem approach, those who have an established and positive relationship with their GP are likely to be at an advantage. Walter *et al. *[13] found that, in existing and positive GP-caregiver relationships, German GPs found it easier to overcome the hurdles of time constraints and diffidence in order to deliver preventive care to older patients. Caregivers who lack such a relationship with their GP might be disadvantaged, although an established relationship cannot guarantee a timely and appropriate response. GPs' capacity to engage effectively with caregivers as patients also depends on their communication skills, experience, perspective and whether they interact with these patients opportunistically. This study suggests that GPs might need to take the initiative with caregivers as patients routinely. An appropriate tool could help caregivers to identify and communicate their needs, and the feasibility and efficacy of two such tools for reducing the levels of unmet needs are currently being tested. The first tool, the Needs Assessment Tool: Progressive Disease - Cancer (NAT:PD-C), enables health professionals to assess the needs of patients with progressive cancer as well as those of their primary caregiver comprehensively, and to do so in a timely and appropriate manner, so that the allocation of palliative care resources can be based on need [25]. This tool is complemented by a set of evidence-based guidelines. The second tool, the Needs Assessment Tool for Caregivers (NAT-C), enables caregivers to proactively self-identify their level of concern with specific aspects of their health and well-being, to prioritize these concerns, then raise with their GP those they wish to discuss [20]. This assessment tool is complemented by a set of GP resources indexed to the tool, and is currently the subject of a randomised controlled trial [20].

### Limitations

Our belief that GPs' care of caregivers as patients is desirable and achievable might have influenced the framing of the interview questions. However, every effort was made to develop questions which were relevant to existing literature and practice norms, and both converging and diverging views have been reported. We recruited six caregivers in twelve weeks, including only one man and only one non-metropolitan caregiver, although we had expected to recruit at least ten. Our experience highlights the challenges of reaching and recruiting caregivers within the constraints of site requirements. As a result of the combined effect of our small caregiver sample size and the potential sensitivity of our research topic, it is unlikely that saturation of themes was reached. However, we believe our limited data have yielded important insights. Multiple participants provided us with evidence which suggests that, for different reasons, both caregivers as patients *and *their GPs often fail to take the initiative regarding caregiver's valid health concerns. We also included exceptions to the pattern, such as the negative reaction to a GP who inquired after the caregiver's health, and the challenge to the GP's finite skills and experience when caregivers as patients deliberately avoid discussing their own legitimate needs. Some participants may have felt inhibited from discussing their views and experiences with a researcher, although the interviews were conducted by a well-experienced interviewer.

### Recommendations

Interventions need to be developed and tested to enhance communication between caregivers as patients and their GPs. The implications of these findings for general practice training also need to be explored. Policy changes are needed to make care of caregivers a more attractive, viable component of general practice, such as appropriate remuneration for assessing and addressing caregivers' own health needs.

## Conclusions

This paper sheds new light on the underlying norms in the paradox whereby the health needs of caregivers as patients can become tangential to the cancer patients' needs instead of being considered and supported. Caregivers may or may not wish to raise their health concerns with their GP, so it may ultimately fall to the GP to take the initiative by cueing caregivers that it is timely and safe for them to do so. The routine use of an accessible prompt may ensure that needs are systematically addressed. The task is limited by GPs' desire and capacity to engage with caregivers as patients, but the potential difference GPs can make to the health of these patients is substantial.

## Abbreviations

GP: General Practitioner; HP: Health professional; RN: Registered Nurse; C: Caregiver; NAT:PD-C: Needs Assessment Tool: Progressive Disease - Cancer; NAT-C: Needs Assessment Tool for Caregivers

## Competing interests

The authors declare that they have no competing interests.

## Authors' contributions

GM, MJ and AG conceived and designed the parent study, and obtained funding. LB and GM conducted the qualitative analysis. LB drafted the paper, and all authors contributed substantively to its development. All authors read and approved the final manuscript.

## Pre-publication history

The pre-publication history for this paper can be accessed here:

http://www.biomedcentral.com/1471-2296/12/110/prepub

## References

[B1] Australian Government. CanNET Information Bulletin No. 2 Needs based approach to cancer 2007 Canberra: Australian Government 8

[B2] World Health Organization Media Centre Cancer Fact sheet No. 297 2011 World Health Organization

[B3] Australian Institute of Health and Welfare Carers in Australia: Assisting frail older people and people with a disability. AIHW Cat No. AGE 41 2004 Canberra: AIHW

[B4] Winer E Gralow J Diller L Karlan B Loehrer P Pierce L Clinical cancer advances 2008: major research advances in cancer treatment, prevention, and screening - a report from the American Society of Clinical Oncology J Clin Oncol 2009 27 812 826 10.1200/JCO.2008.21.2134 19103723 PMC2645086

[B5] Briggs H Fisher D Warning: caring is a health hazard: results of the 1999 national survey of carer health and wellbeing 2000 Canberra: Carers Association of Australia

[B6] Pinquart M Sorensen S Differences between caregivers and noncaregivers in psychological health and physical health: a meta-analysis Psychol Aging 2003 18 250 267 12825775 10.1037/0882-7974.18.2.250

[B7] Morris S Thomas C The carer's place in the cancer situation: where does the carer stand in the medical setting? Eur J Cancer Care (Engl) 2001 10 87 95 10.1046/j.1365-2354.2001.00249.x 11829054

[B8] Jones RVH Hansford J Fiske J Death from cancer at home: the carers' perspective Br Med J 1993 306 249 251 10.1136/bmj.306.6872.249 PMC1676719 8443527

[B9] Bruce D Paley G Underwood P Roberts D Steed D Communication problems between dementia carers and general practitioners: effect on access to community support services Med J Aust 2002 177 186 188 12175321 10.5694/j.1326-5377.2002.tb04729.x

[B10] Bulsara C Fynn N An exploratory study of GP awareness of carer emotional needs in Western Australia BMC Fam Pract 2006 7 33 10.1186/1471-2296-7-33 16719930 PMC1479827

[B11] Fox F Rodham K Harris M G T Sutton J Scott J Robinson B Experiencing "The Other Side": A Study of Empathy and Empowerment in General Practitioners Who Have Been Patients Qual Health Res 2009 19 1580 1588 10.1177/1049732309350732 19843966

[B12] Council on Scientific Affairs AMA Physicians and family caregivers: a model for partnership JAMA 1993 269 1282 1284 8437307

[B13] Walter U Flick U Neuber A Fischer C Hussein R Schwartz F Putting prevention into practice: qualitative study of factors that inhibit and promote preventive care by general practitioners, with a focus on elderly patients BMC Fam Pract 2010 11 68 10.1186/1471-2296-11-68 20854654 PMC2949669

[B14] Wade D Halligan P Do biomedical models of illness make for good healthcare systems? Br Med J 2004 329 1398 1401 10.1136/bmj.329.7479.1398 PMC535463 15591570

[B15] Stewart M Towards a global definition of patient centred care: the patient should be the judge of patient centred care Br Med J 2001 322 444 445 10.1136/bmj.322.7284.444 PMC1119673 11222407

[B16] Levenstein J McCracken E McWhinney I Stewart M Brown J The Patient-Centred Clinical Method. 1. A Model for the Doctor-Patient Interaction in Family Medicine Fam Pract 1986 3 24 30 10.1093/fampra/3.1.24 3956899

[B17] Little P Everitt H Williamson I Warner G Moore M Gould C Preferences of patients for patient centred approach to consultation in primary care: observational study Br Med J 2001 322 1 7 10.1136/bmj.322.7277.1 PMC26564 11222423

[B18] Garfinkel H Studies in Ethnomethodology 1967 Englewood Cliffs: Prentice Hall

[B19] Patton MQ Qualitative Research & Evaluation Methods 2002 3 Thousand Oaks, London, New Delhi: Sage Publications21980612

[B20] Mitchell G Girgis A Jiwa M Sibbritt D Burridge L A GP Caregiver Needs Toolkit versus usual care in the management of the needs of caregivers of patients with advanced cancer: a randomized controlled trial Trials 2010 11 115 10.1186/1745-6215-11-115 21114863 PMC3009964

[B21] Green J Thorogood N Qualitative Methods for Health Research 2004 London: Sage Publications Ltd

[B22] Nolan M Grant G Keady J Understanding Family Care: a Multidimensional Model of Caring and Coping 1996 Buckingham UK, Philadelphia USA: Open University Press

[B23] Silverman D Going private: ceremonial forms in a private oncology clinic Sociology 1984 18 191 204

[B24] Payne S Smith P Dean S Identifying the concerns of informal carers in palliative care Palliative Medicine 1999 13 37 44 10.1191/026921699666872524 10320874

[B25] Waller A Girgis A Johnson CGM Yates P Kristjanson L Facilitating needs based cancer care for people with a chronic disease: Evaluation of an intervention using a multi-centre interrupted time series design BMC Palliative Care 2010 9 2 10.1186/1472-684X-9-2 20150987 PMC2820447

